# Appetitive and reactive aggression are differentially associated with the STin2 genetic variant in the serotonin transporter gene

**DOI:** 10.1038/s41598-018-25066-8

**Published:** 2018-04-30

**Authors:** Sian Megan Joanna Hemmings, Khethelo Xulu, Jessica Sommer, Martina Hinsberger, Stefanie Malan-Muller, Gerard Tromp, Thomas Elbert, Roland Weierstall, Soraya Seedat

**Affiliations:** 1 Department of Psychiatry, Faculty of Medicine and Health Sciences, Stellenbosch University, Tygerberg, South Africa; 2 Department of Psychology, University of Konstanz, Konstanz, Germany; 3 Division of Molecular Biology and Human Genetics, Faculty of Medicine and Health Sciences, Stellenbosch University, Tygerberg, South Africa; 4 Clinical Psychology and Psychotherapy, Medical School Hamburg, Hamburg, Germany

## Abstract

Appetitive aggression is a sub-category of instrumental aggression, characterised by the primary intrinsic enjoyment of aggressive activity. Aggression is heritable, and serotonergic and monoaminergic neurotransmitter systems have been found to contribute to the underlying molecular mechanisms. The aim of this study was to investigate the role that genetic variants in the serotonin transporter (*SLC6A4*) and monoamine oxidase A (*MAOA*) genes play in the aetiology of appetitive aggression in South African Xhosa males (n = 290). *SLC6A4* 5-HTTLPR, rs25531, and STin2 variants, as well as *MAOA*-uVNTR were investigated for their association with levels of appetitive aggression using Poisson regression analysis. The STin2 VNTR12 allele was found to be associated with increased levels of appetitive aggression (p = 0.003), but with decreased levels of reactive aggression (p = 7 × 10^−5^). This study is the first to investigate genetic underpinnings of appetitive aggression in a South African population, with preliminary evidence suggesting that *SCL6A4* STin2 variants play a role in its aetiology, and may also be important in differentiating between appetitive and reactive aggression. Although the results require replication, they shed some preliminary light on the molecular dichotomy that may underlie the two forms of aggression.

## Introduction

Aggression can occur in two main forms: appetitive and reactive aggression, which are broadly characterised by the motivation to perpetrate aggression^1^. Appetitive aggression is driven by the purpose of attaining social status and the violent self-rewarding lust and enjoyment of inflicting pain through violence^1^. Conversely, reactive aggression is a reactive, emotional response, usually occurring after provocation or in response to a life-threatening situation^1,2^.

Although the environment contributes to the development of aggression, studies indicate that genetic factors explain up to 65% of the variability in violent, aggressive and impulsive behaviour^3–7^. Interestingly, the heritability of aggressive traits has been found to increase with age. In a recent meta-analysis, Burt *et al*.^3^ noted an increase in heritability from 55% at ages 1–5 years to 65% at 11–18 years of age. Moreover, heritability of aggressive traits has been shown to be higher in males compared to females^6,8,9^.

The majority of genetic association studies in aggression have employed a candidate gene approach, whereby genes are selected for investigation based on the current knowledge regarding the biology of the disorder or trait. Numerous lines of evidence indicate that low or impaired serotonin function underlies the traits of aggression and impulsivity^10–15^. Serotonin may be involved in the withdrawal from dangerous or aversive situations, thus a hypofunctioning of the serotonergic system (the so-called “serotonin deficiency hypothesis”) could result in impaired avoidance of aversive stimuli or undesirable situations, and could lead to impulsive, aggressive and violent behaviour and responses^16,17^.

The serotonin transporter (also known as solute carrier family 6 member 4 (SLC6A4)) plays an important role in the regulation of serotonin concentration in the brain^18^. The gene encoding SLC6A4 is located on chromosome 17q11.1–q12^19^, and has been extensively investigated in the context of anxiety and depressive disorders. The gene contains an insertion-deletion (indel) polymorphism known as the serotonin transporter-linked polymorphic region (5-HTTLPR)^19,20^. This polymorphism comprises a short (S) allele and a long (L) allele, which differ in length by 44 base pairs (bp). 5-HTTLPR is reportedly functional, with the L-allele facilitating more efficient transcription than the S-allele^19,21–23^. Recently, Hu *et al*.^24^ identified a single nucleotide polymorphism (SNP), rs25531, situated within the 5-HTTLPR variant. This SNP has been found to modulate the functionality of the L-allele, such that the L-G haplotype results in reduced *SLC6A4* expression (comparable to that of the S-allele), whilst the L-A haplotype is associated with increased *SLC6A4* expression, and increased SLC6A4 binding potential in the putamen^25^. Many researchers, therefore, have indicated that it is functionally more sound to group subjects who carry the L-G haplotype with those carrying S-alleles^24,26^. This is particularly important in populations with African ancestry, as the frequency of the L-G haplotype has been found to be much higher than in other populations^27^.

Another *SLC6A4* variant that has been investigated for its role in aggression is a variable number of tandem repeats (VNTR) polymorphism located in intron 2, referred to as STin2. This polymorphism comprises various repeats of a 17 bp motif, with the most common alleles represented by the 9-repeat (STin2.9), 10-repeat (STin2.10) and 12-repeat (STin2.12) variants^20^. Studies have found that STin2.12 enhances transcription of *SLC6A4*^19,28–30^, and STin2.10 has been associated with less efficient serotonin turnover^30,31^. The STin2.12 allele has been found to be associated with increased aggression in children^32,33^ and adults^34,35^, although association between STin2.12 allele and aggression in the latter two studies was observed only in combination with at least one 5-HTTLPR S-allele. Indeed, recent evidence suggests that the 5-HTTLPR and STin2 VNTR polymorphisms may be controlled by the same regulatory pathway^36^, with the S-STin2.12 allele combination resulting in increased *SLC6A4* expression compared to the L-STin2.10 allele combination.

The monoamine oxidase A (MAOA) enzyme is responsible for degradation of serotonin, dopamine and norepinephrine, and thus plays an important role in the regulation of levels of these neurotransmitters. The gene encoding MAOA is located on the X chromosome, and has long been associated with aggression. Brunner *et al*.^37^ observed that a *MAOA* point mutation in exon 8 was associated with Brunner Syndrome, characterised by increased antisocial behaviour, aggression, and very high levels of disruptive and violent outbursts in affected males, in a large Dutch kindred. Subsequent studies found that adult male mice lacking *MAOA* exons 2 and 3 (resulting in MAOA deficiency) exhibited significantly increased levels of aggressive behaviour^38^. These results are in line with those observed in a mouse model with a novel, spontaneous nonsense mutation in exon 8 (effectively resulting in *MAOA* deletion)^39^. More recently, Palmer *et al*.^40^ observed, amongst others, episodic explosive aggression in individuals with *MAOA* loss-of-function mutations.

*MAOA* contains a polymorphism located in the promoter region, approximately 1.2 kilobases (kb) upstream of the coding region^41^. This VNTR polymorphism (*MAOA*-uVNTR) is characterised by variable numbers of 30 bp repeats, and commonly comprises 2-, 3-, 3.5-, 4- and 5-repeat alleles^42–44^. The *MAOA*-uVNTR 2- and 3-repeat alleles (termed *MAOA*-L in the current manuscript) are associated with reduced transcriptional efficiency compared to the 3.5- and 4-repeat alleles (termed *MAOA*-H in the current manuscript)^43–47^. The functionality of the 5-repeat allele is not clear^43^. Evidence abounds for a sexually-dimorphic role of the *MAOA-*uVNTR in aggression. Males possessing the low-activity alleles have been found to be at greater risk for increased aggressive and impulsive reactions to stressful stimuli, whilst females carrying the high-activity alleles have been found to possess an increased risk of aggression, but only if they have also been exposed to increased levels of early adversity (reviewed in Godar *et al*.^48^).

South Africa is characterised by social and economic inequalities, with poorer communities experiencing daily traumatic stressors. Currently the rates of homicide, gender-based violence and gang-related violence in South Africa are amongst the highest in the world^49^. Exposure to stressors results in the activation of biological and psychological responses that are necessary for adaptation to the environment. Repeated and prolonged exposure to violence and stressful events can, however, result in sustained activation of biologically-mediated responses, with the result that the individual becomes susceptible to an array of both physical and psychological complications^50,51^. Early childhood abuse and adversity, which coincides with the cycle of violence, facilitates the development of violent behaviour and cruelty^52^.

The current study follows on from a recent study by Hinsberger *et al*.^53^, who investigated attraction to violence in the context of continuous traumatic stress exposure in the same study sample originating from townships in Cape Town, South Africa. Here, it was found that appetitive aggression scores were predicted by witnessed as well as self-experienced traumatic events. In the current study, we aimed to investigate, in an exploratory study, whether genetic variants in *SLC6A4* and/or *MAOA* accounted for, at least partially, some of the remaining variance in appetitive aggression score, after correcting for severity of witnessed and self-experienced traumatic events.

## Results

### Clinical data

All participants had experienced at least one type of trauma, with the maximum type of traumas experienced being 16^53^. On average, participants witnessed 10.2 (SD = 2.6) traumatic events. The average number of self-experienced trauma types was 8.4 (SD = 3.0). The AAS scores ranged from 0 to 60, with a median of 12 (IQR: 6–23.5)^53,54^.

The AAS total score was found to be negatively correlated with age (r = −0.11; p = 0.07), although this was not statistically significant. As in Hinsberger *et al*.^53^, both self-experienced trauma and witnessed trauma were found to be positively correlated with AAS total score (r = 0.38 [p < 0.001] and r = 0.32 [p < 0.001], respectively).

The mean BPAQ score was 86.2 (SD = 20.3), and was not significantly correlated with age (r = 0.03; p = 0.52). However, AAS and BPAQ scores were highly correlated with one another (r = 0.55; p < 2 × 10^−16^). In addition, both self-experienced and witnessed trauma were positively correlated with BPAQ score (r = 0.38 [p < 0.001] and r = 0.32 [p < 0.001], respectively).

### Genetic association results

All genotype calls were 100% concordant with sequencing results. Genotypes for STin2 and *MAOA-*uVNTR genotypes were in HWE (p = 0.734 and p = 1.0, respectively). The genotypes for 5-HTTLPR and rs25531 were not in HWE (p < 0.001 for both variants), and these were thus not investigated any further. Genotype distribution summaries for the variants are provided in Table 1.

**Table 1:** Genotype counts and frequencies (%) of *SLC6A4* and *MAOA* variants. P-values are from exact tests of Hardy-Weinberg equilibrium.

Variant	Genotype	Count (%)	HWE (p-value)
rs25531	AA	209 (72.1)	<0.001
	AG	50 (17.2)	
	GG	31 (10.7)	
*5-*HTTLPR	LL	122 (42.1)	<0.001
	LS	160 (55.2)	
	SS	8 (2.8)	
*5*-HTTLPR*-*rs25531	L’L’	86 (29.7)	<0.001
	L’H’	115 (39.7)	
	H’H’	89 (30.6)	
STin2^1^	12/12	174 (60.0)	0.734
	12/10	103 (35.5)	
	10/10	13 (4.5)	
*MAOA-*uVNTR	*MAOA-*L	136 (49.4)	0.660^4^
	*MAOA-*H	139 (50.5)	

Abbreviations: *5-*HTTLPR: serotonin transporter-linked polymorphic region; *MAOA:* monoamine oxidase A; HWE: Hardy-Weinberg equilibrium.

^1^Alleles are represented as repeats: 12 repeat = 301 bp (high expressing allele); 10 repeat = 267 bp (low-expressing allele).

^2^*MAOA*-L: *MAOA*-uVNTR low-expressing allele (2-repeat + 3-repeat alleles).

^3^*MAOA-*H*: MAOA-*uVNTR high-expressing allele (4-repeat + 5-repeat alleles).

^4^HWE was calculated based on ungrouped *MAOA* allele frequencies; individual frequencies are as follows: 291 bp (2-repeat) = 19 (6.9%), 321 bp (3-repeat) = 117 (42.5%), 351 bp (4-repeat) = 134 (48.7%), 381 bp (5-repeat) = 5 (1.8%).

The known covariates of appetitive aggression (experienced and witnessed trauma) in the current sample^53^, as well as age, BPAQ score and each of the genetic variants (additive inheritance model) were regressed on AAS total score, using Poisson regression (Tables 2 and 3). All models were found to be good fits for the data, using the Hosmer-Lemeshow goodness-of-fit approach implemented in the R package “*ResourceSelection*”^55,56^.

**Table 2:** Association between *SLC6A4* STin2 variant and appetitive aggression (AAS score), using additive genetic models.

Variable	Unstandardised coefficient		z	p	95% CI for β	
	β	SE			Upper bound	Lower bound
Intercept	0.95	0.13	7.59	3.23 × 10^−14^	0.70	1.19
Witnessed trauma	0.03	0.01	4.65	3.39 × 10^−06^	0.02	0.05
Experienced trauma	0.06	0.06	8.19	2.59 × 10^−16^	0.04	0.07
Age (years)	−0.04	0.00	−10.04	<2 × 10^−16^	−0.05	−0.03
BPAQ score	0.02	0.00	21.77	<2 × 10^−16^	0.02	0.02
STin2.12	0.08	0.03	3.17	0.0015*	0.03	0.13

Abbreviations: β, estimated Poisson regression coefficients for the model; SE, standard error of the individual regression coefficients; z, z-test statistic; CI, confidence interval; BPAQ, Buss-Perry Aggression Questionnaire; BPAQ, Buss-Perry Aggression Questionnaire; STin2.12, 12-repeat allele of the serotonin transporter intron 2 variant.

Hosmer-Lemeshow goodness-of-fit: χ^2^ = −3.95, df = 8, p-value = 1.

*Benjamini-Hochberg corrected p = 0.003.

**Table 3:** Association between *MAOA-*uVNTR variant and appetitive aggression (AAS score), using additive genetic models.

Variable	Unstandardised coefficient		z	p-value	95% CI for β	
	β	SE			Upper bound	Lower bound
Intercept	1.12	0.12	9.71	<2 × 10^−16^		
Witnessed trauma	0.04	0.01	5.21	1.92 × 10^−07^	0.02	0.05
Experienced trauma	0.05	0.01	7.13	9.80 × 10^−13^	0.04	0.07
Age (years)	−0.04	0.00	−9.82	<2 × 10^−16^	−0.05	−0.03
BPAQ score	0.02	0.00	20.53	<2 × 10^−16^	0.02	0.02
*MAOA-*L	0.01	0.03	0.35	0.728	−0.05	0.07

Abbreviations: β, estimated Poisson regression coefficients for the model; SE, standard error of the individual regression coefficients; z, z-test statistic; CI, confidence interval; BPAQ, Buss-Perry Aggression Questionnaire; *MAOA*, monoamine oxidase A; *MAOA*-L: low-expressing *MAOA-*uVNTR allele combination (2- and 3-repeat alleles).

Hosmer-Lemeshow goodness-of-fit: χ^2^ = −4.95, df = 8, p-value = 1.

The STin2 VNTR was found to be significantly associated with AAS score (corrected p = 0.003), with the addition of each STin2.12 allele increasing the AAS score by a total of 9% (exp[0.082] = 1.09). *MAOA* uVNTR was not found to be associated with appetitive aggression in the present study (p = 0.728) (Table 2).

When reactive aggression was investigated, we found that the STin2 VNTR was significantly associated with BPAQ score; however, this association was in the opposite direction to that observed for AAS score. With each addition of the STin2.12 allele, the BPAQ was found to reduce by 5% (exp[−0.046] = 0.095) (corrected p = 7 × 10^−5^) (Table 4). No significant association was observed between *MAOA*-uVNTR and BPAQ score (p = 0.123) (Supplementary Table S1).

**Table 4:** Association between STin2 VNTR variant and reactive aggression (BPAQ score), using additive genetic models.

Variable	Unstandardised coefficient		z	p	95% CI for β	
	β	SE			Upper bound	Lower bound
Intercept	4.21	0.04	100	<2 × 10^−16^	0.70	1.19
Witnessed trauma	0.003	0.003	0.88	0.38	−0.003	0.009
Experienced trauma	0.02	0.003	6.72	0.0000	0.014	0.025
Age (years)	−6.8 × 10^−6^	0.002	−0.004	0.9960	−0.003	0.003
AAS score	0.008	0.0005	14.54	<2 × 10^−16^	0.007	0.009
Stin2.12	−0.05	0.011	−4.30	1.73 × 10^−5^*	−0.07	−0.03

Abbreviations: β, estimated Poisson regression coefficients for the model; SE, standard error of the individual regression coefficients; z, z-test statistic; CI, confidence interval; BPAQ, Buss-Perry Aggression Questionnaire; BPAQ, Buss-Perry Aggression Questionnaire; STin2.12, 12-repeat allele of the serotonin transporter intron 2 variant.

Hosmer-Lemeshow goodness-of-fit: χ^2^ = −207.95, df = 8, p-value = 1.

*Benjamini-Hochberg corrected p-value = 7 × 10^−5^.

## Discussion

This is the first study to investigate the relationship between the *SLC6A4* and *MAOA* genes and appetitive aggression in a South African male population of Xhosa ethnicity. Serotonin has long been implicated in the aetiology of aggression^57^, and numerous publications have investigated the association between serotonergic genes and various forms of aggression. However, appetitive aggression has been largely excluded from these investigations.

We observed a significant association between the STin2 VNTR variant and levels of appetitive aggression, measured using the AAS^58^. Here, the STin2.12 repeat allele was found to increase the AAS score by 9% (p = 0.003). It is interesting to note that, while STin2 VNTR was associated with reactive aggression in our population as well, the association was found to be in the opposite direction, with the STin2.12 allele reducing BPAQ score by 5% (p = 7 × 10^−5^). Aggression is a heterogeneous multi-dimensional construct, which can be very broadly divided into reactive (hostile-affective) and proactive (instrumental-predatory) aggression^59,60^. Appetitive aggression is a form of proactive aggression, which is defined as goal-oriented, proactive and controlled^1,61^. Reactive aggression, on the other hand, occurs in response to a perceived threat. Although the value of distinguishing between the two types of aggression has been debated^1,62^, the differentiation may have implications regarding intervention, diagnosis and prevention^63^. Indeed, unique risk factors have been found to be associated with both types of aggression, and differences in serotonergic functioning may partially underlie the dichotomy. Our results provide the first evidence that the two forms of aggression can be differentiated by *SLC6A4* STin2 genetic variants.

Genes containing the STin2.12 allele have been found to have higher rates of *SLC6A4* transcription compared to those with the STin2.10 allele^29,30,64^. Increased rates of *SLC6A4* transcription would, theoretically, result in more efficient clearing and thus reduced availability of serotonin in the synaptic cleft. Serotonin is a key neurotransmitter in the central nervous system, and has been found to be important in numerous brain functions, including neurogenesis, apoptosis and synaptic plasticity. Alterations in serotonin concentrations could thus have consequences for brain function and behaviour^65^. In line with the currently debated serotonin deficiency hypothesis of human aggression, reduced serotonergic tone has been found to be associated with increased risk for pathological aggression^10,14,66,67^, although a recent hypothesis by Montoya *et al*.^68^ suggests that it is the ratio of testosterone to cortisol that predisposes one to aggressive behaviour, and the levels of serotonin that tip the scale in favour of either reactive (low serotonin levels) or instrumental (high serotonin levels) aggression. Given the latter hypothesis, our present results are notable, in that we observed an association between STin2.12 (associated with lower levels of serotonin) and appetitive aggression, with the opposite effect observed for reactive aggression. It is, however, important to keep in mind that serotonergic regulation in the central nervous system is highly complex – factors modifying the regulation of serotonin (including genes and environment) will vary between study populations, and depending on interaction between the modifiers, may result in association between variables that differ between studies. What these results do indicate, however, is that there seems to be a U-shaped “Goldilocks” effect, where either too much or too little serotonin is associated with either reactive or appetitive aggression, and may therefore may be useful in differentiating between the two forms of aggression.

Literature abounds regarding the association of the *MAOA-*uVNTR low-expressing alleles and aggression^69–71^. However, no significant association was observed between *MAOA*-uVNTR and either appetitive or reactive aggression. The lack of evidence of association between *MAOA-*uVNTR and appetitive aggression in our study may be due to a number of factors, including a lack of power to detect possibly small effects, lack of LD of the *MAOA-*uVNTR with the actual causal variant, and a difference in study designs.

The lack of HWE for the 5-HTTLPR and rs25531 variants is interesting, although perhaps unsurprising in the present context, given the sample used in the investigation. Deviation from HWE may indicate, amongst others, population stratification, inbreeding or genotyping error. For HWE to be fulfilled, a number of assumptions, including random mating, lack of selection according to genotype and absence of mutation or migration should be met. It is unlikely that deviation from HWE in the present study is due to genotyping error, as we validated genotype results by sequencing a randomly selected 10% of the sample for all the variants investigated. We did not formally test for population stratification in our sample; however, all participants were of Xhosa ethnicity, a group of predominantly Bantu-speaking individuals in South Africa. The Xhosa population is currently the second-largest ethnic group in the country, constituting approximately 18% of the South African population^72^. Although no in-depth analysis has been performed to study the underlying genetic substructure in the Xhosa population, the Niger-Kordafarian linguistic subgroup to which they belong has been found to exhibit relative genetic homogeneity^73–75^. In addition, the Xhosa population is also characterised by cultural and ethnic isolation and thus less likely to present with genetic and phenotypic heterogeneity^76^. Deviations from HWE in the present study are thus likely to be the result of selection bias. Participants were a highly select group of males recruited from two low-socioeconomic areas, in the Western Cape, known for high levels of violence and PTSD^49^. Recruitment focussed on males who were former young offenders (identified via a reintegration programme) or were at risk of perpetrating crimes (recruited via police stations or concerned family members)^53,54^. The 5-HTTLPR and rs25531 variants may thus be associated with an as yet uninvestigated trait in this specific population, causing HWE to deviate significantly.

The current study is a novel one, which yields interesting findings. However, the results should be interpreted in the context of some important limitations. First, the specificity of the population in the current study invalidates generalisation to other populations, necessitating replication of the results in samples recruited from the general population. Second, as mentioned above, we did not correct for population stratification in our Xhosa sample. Although there is historical and cultural evidence to suggest that the Xhosa population may be, for all intents and purposes, genetically homogenous, this needs to be tested empirically, in a sample with sufficient power to provide the required resolution. In addition, the effect sizes for each of the significant genetic associations reported are small, necessitating replication studies using increased numbers of samples to attain sufficient power to identify robust associations between STin2 and appetitive and reactive aggression.

The study represents the first to investigate the association between genetic variants in *SLC6A4* and *MAOA* and appetitive aggression in male South Africans of Xhosa ethnicity who were identified as being at high risk for perpetrating violence. Although the selected variants have been widely studied in reactive or impulsive types of aggressive behaviour, the current study is the first to investigate 5-HTTLPR, STin2 and *MAOA*-uVNTR polymorphisms in the context of appetitive aggression. We provide, for the first time, preliminary evidence suggesting that STin2 VNTR may be important in distinguishing appetitive from reactive aggression. However, given the aforementioned limitations, the present results should be interpreted with caution until such time as they are replicated in a scientifically rigorous manner.

## Methods

### Ethical considerations

The study was approved by the ethics review boards of University of Konstanz, Stellenbosch University and University of Cape Town. Participants received compensation for taking part in the study. All research was performed in accordance with relevant guidelines and regulations. All participants over the age of 18 years gave informed consent to participate in the study, and informed consent was obtained from parents or caretakers for participants under the age of 18 years.

### Clinical and demographic information

The cohort of 290 male Xhosa participants were all recruited from the townships of Khayelitsha and Gugulethu in the Western Cape, South Africa. “Township” in South Africa refers to an underdeveloped urban residential area that was historically reserved for non-white inhabitants. The socio-economic conditions of most townships in South Africa are poor, and unemployment rates are very high.

All participants were recruited through a reintegration centre for offenders and youth deemed to be at risk of experiencing and perpetrating violence due to high levels of gang violence and substance abuse present in the low-income communities of Cape Town in which the participants lived. The final sample comprised those individuals who were attending a reintegration program at the time (51%) and those who had not previously participated in a reintegration program (49%).

Sociodemographic information was obtained from each participant and included age and educational background^53,54^. Participants ranged in age between 14 and 40 years, with a median of 21 years (IQR: 19–24 years). All participants were of isiXhosa ethnicity, and the majority (80.7%; n = 238) had not completed high school. Of the 19.3% who had completed high school, four (1.4%) attended college^53^.

Trauma exposure was measured using an adapted Childhood Exposure to Community Violence Checklist (CECV)^77^. The CECV is a 33-item self-report checklist that assesses children’s levels of witnessing, experiencing or hearing about trauma. The questionnaire was adapted to reflect types of violence typical of low-income areas in South Africa, such as sexual and physical assault. The CECV has been used previously in South African populations^78^ and offender populations^79^. The events can be categorised as either “witnessed” or “experienced”. The total score on the CECV indicates an individual’s severity of exposure to traumatic events and community violence. The reliability of the CECV score in the current sample, measuring internal consistency using the McDonald’s coefficient omega, has been found to be 0.79 (95% CI: 0.75–0.82)^54^.

Appetitive aggression was measured using the Appetitive Aggression Scale (AAS)^58^. Here, responses to 15 questions on instrumental aggression, addiction behaviour and desire to do harm were rated on a 5-point Likert scale, and the total AAS score calculated by summing the scores of the 15 items. The reliability of the score has been found to be high in the current sample (McDonald’s coefficient omega = 0.87; 95 CI: 0.84–0.89)^54^.

Reactive aggression was measured using the Buss-Perry Aggression Questionnaire (BPAQ) score^80^. The BPAQ is a 29-item inventory scored on a 5-point Likert-type scale from 1 to 5. Higher scores on the BPAQ indicate higher levels of trait aggression.

### Genotyping methods

Genomic DNA was extracted from saliva collected in Oragene^TM^ DNA self-collection kits (OG-500, DNA Genotek, Ontario, Canada) using the Prep-It L2P reagent (DNA Genotek, Ontario, Canada) as per manufacturer’s instructions. The 5-HTTLPR and rs25531 polymorphisms were genotyped by employing a two-stage genotyping procedure, as previously described^81^. The STin2 VNTR polymorphism was amplified using previously published primer sequences adapted from Battersby *et al*.^82^. The forward primer was fluorescently labelled with the fluor HEX, to facilitate genotyping by capillary electrophoresis. The 20 µl PCR reaction mixture comprised 12.5 µl KAPA ReadyMix (Kapa Biosystems, Wilmington, MA, USA), 0.15 µM each of the forward and reverse primers, and 20 ng template DNA, made up to a final volume of 20 µl using bi-distilled water. PCR conditions were as follows: an initial 5-min denaturation step at 95 °C, 35 cycles of 95 °C for 60 s, 60 °C for 30 s, and 72 °C for 45 s and a final 7-min extension step at 72 °C. PCR amplicons were separated by agarose gel electrophoresis and visualized by ethidium bromide staining to assess the PCR success.

The loci containing the 5-HTTLPR/rs25531, and STin2 VNTR polymorphisms were amplified separately. Following agarose gel electrophoresis to determine the success of each PCR, the amplicons (STin2 and 5*-*HTTLPR) were combined in a 1:1 ratio for capillary electrophoresis.

*MAOA*-uVNTR amplification was performed using published primer sequences adapted from Sabol *et al*.^44^, which generates PCR amplicons of sizes 291 bp (2-repeat allele), 321 bp (3-repeat allele), 336 bp (3.5-repeat allele), 351 bp (4-repeat allele), and 381 bp (5-repeat allele). The forward primer was fluorescently labelled using VIC^TM^ in order to facilitate capillary electrophoresis. For statistical analyses, *MAOA-*uVNTR alleles were grouped according to low activity (2-repeat [291 bp] and 3-repeat [321 bp] alleles) and high-activity (4-repeat [351 bp] and 5-repeat [381 bp]) alleles.

Capillary electrophoresis was performed on an ABI Prism 3730 Genetic Analyzer (Applied Biosystems, Foster City, CA, USA) at the Central Analytical Facility (CAF-SUN Unit, Stellenbosch, South Africa).

To verify the genotypes from each variant investigated, 10% of the sample was randomly selected, PCR amplified and sequenced using primers flanking each of the variants. The sequences were analysed using the BIOEdit software (http://www.mbio.ncsu.edu/BioEdit/bioedit.html).

### Statistical analysis

Hinsberger *et al*.^53^ previously conducted a path analysis in order to investigate the role of attraction to violence in the context of ongoing stress, and found that attraction to violence was predicted by the witnessed traumatic events as well as victimisation^53^. The aim of the present study was to identify genetic variants associated with appetitive aggression, and because instrumental/proactive aggression is usually significantly correlated with reactive aggression^58^, we included the BPAQ score in the regression model as a covariate.

The outcome data (level of appetitive aggression, assessed by means of the AAS score) were distributed as a bounded rare event, i.e., a Poisson-like distribution. Therefore, we regressed AAS score against genotype, adjusting known AAS covariates in this sample, namely witnessed and self-experienced trauma^53^, as well as age and BPAQ score. Poisson regression provides a model that describes how the mean value of the response variable (λ), changes as a function of one or more explanatory variables. In the glm link model for variables that possess a Poisson distribution, we assume that the ln(λ) is linearly related to the independent variables in the following manner:

$$\mathrm{ln}(\lambda )={\beta }_{0}+{\beta }_{1}{x}_{1}+{\beta }_{2}{x}_{2}+{\beta }_{3}{x}_{3}+{\beta }_{4}{x}_{4}+{\beta }_{5}{x}_{5}$$

where λ represents the mean AAS score, *β*_0_ the model intercept, and *x*_1_ to *x*_5_ represents each of the explanatory variables, namely witnessed trauma, experienced trauma, age (years), BPAQ score and either Stin2 VNTR or *MAOA* genotype, with their associated parameter estimates (*β*_1_ to *β*_5_). The exponentiated β estimate is thus the multiplicative term used to calculate predicted AAS scores when the explanatory variable increases by one unit.

All analyses were performed using R v3.2.2 and base functions of R^83^. Multiple testing correction was implemented using Benjamini-Hochberg false discovery rate (FDR)^84^. Goodness-of-fit tests were performed using the Hosmer-Lemeshow test^85^.

### Data availability

Genotyping data and relevant clinical data will be available from the corresponding author on request.

## Electronic supplementary material

Supplementary Table S1
