# Supplementary material for: Appetitive and reactive aggression are differentially associated with the STin2 genetic variant in the serotonin transporter gene
Source: Sci Rep. 2018 Apr 30;8:6714. doi: 10.1038/s41598-018-25066-8 (PMC5928100; doi:10.1038/s41598-018-25066-8)
Supplement: Supplementary file 1 — Supplementary Table S1 [file 41598_2018_25066_MOESM1_ESM.pdf]

Appetitive and reactive aggression are differentially associated with the STin2 variant in the serotonin transporter gene

Sian Megan Joanna Hemmings*[1], PhD, Khethelo Xulu[1], MSc, Jessica Sommer[2], PhD, Martina Hinsberger[2], PhD, Stefanie Malan-Muller[1], PhD, Gerard Tromp[3], PhD, Thomas Elbert[2], PhD, Roland Weierstall[2,4], PhD, Soraya Seedat[1], PhD

1 Department of Psychiatry, Faculty of Medicine and Health Sciences, Stellenbosch University, South Africa

2 Department of Psychology, University of Konstanz, Reichenau, Konstanz, Germany

Martina Hinsberger

3 Division of Molecular Biology and Human Genetics, Faculty of Medicine and Health Sciences, Stellenbosch University, South Africa

4 Clinical Psychology and Psychotherapy, Medical School Hamburg, Hamburg, Germany

Corresponding author: SMJ Hemmings, Department of Psychiatry, Faculty of Medicine and Health Sciences, Stellenbosch University, PO Box 241, Cape Town, 8000, South Africa

Tel: +27 21 938 9695

Fax: +2721 933 5790

e-mail: smjh@sun.ac.za

Running title: Genetics and appetitive aggression

SUPPLEMENTARY TABLE

Table S1. Association between *MAOA* uVNTR variant and reactive aggression (BPAQ score), using an additive genetic model

**Variable**	**Unstandardized coefficient**		**z**	**p**	**95% CI for β**	
	β	SE			Upper bound	Lower bound
Intercept	4.11	0.04	105.44	$< 2 \times 10^{-16}$	4.04	4.19
Witnessed trauma	0.003	0.003	1.02	0.3070	–0.003	0.01
Experienced trauma	0.02	0.003	7.21	5.53×10^{-13}	0.02	0.03
Age (years)	–0.0004	0.002	–0.23	0.8210	–0.004	0.003
AAS score	0.008	0.0005	13.67	$< 2 \times 10^{-16}$	0.006	0.008
MAOA-L	0.02	0.01	1.54	0.123	–0.006	0.05

Abbreviations: β, estimated Poisson regression coefficients for the model; SE, standard error of the individual regression coefficients; z, z-test statistic; CI, confidence interval; BPAQ, Buss-Perry Aggression Questionnaire; *MAOA*, monoamine oxidase A; *MAOA*-L: low-expressing *MAOA*-uVNTR allele combination (2- and 3-repeat alleles)

Hosmer-Lemeshow goodness-of-fit: $\chi 2$ = -197.26, df = 8, p-value = 1
